# A study of starch resources with high-amylose content from five Chinese mutant banana species

**DOI:** 10.3389/fnut.2022.1073368

**Published:** 2022-12-05

**Authors:** Bo Li, Baoguo Xie, Jin Liu, Xiaoai Chen, Yanjun Zhang, Lehe Tan, Yitong Wang, Libin Zhu, Kexue Zhu, Chongxing Huang

**Affiliations:** ^1^Chinese Academy of Tropical Agricultural Sciences, Spice and Beverage Research Institute, Wanning, Hainan, China; ^2^College of Light Industry and Food Engineering, Guangxi University, Nanning, Guangxi, China; ^3^Key Laboratory of Processing Suitability and Quality Control of the Special Tropical Crops of Hainan Province, Wanning, Hainan, China; ^4^Reproductive Medicine Center, The First Affiliated Hospital of Hainan Medical University, Haikou, China; ^5^Women's and Children's Hospital of Wanning, Wanning, Hainan, China; ^6^School of Forest, Northeast Forestry University, Haerbing, Heilongjiang, China; ^7^College of Food Science, Heilongjiang Bayi Agricultural University, Daqing, Heilongjiang, China

**Keywords:** high amylose banana starch, new Chinese banana resources, A-or B-type crystallize, physicochemical properties, particle morphology properties, statistical analysis

## Abstract

Investigation on staple crop starch of new species has been becoming the research focus of scholars at present. Based on this, the physicochemical properties and microstructural characteristics of starches isolated from Chinese mutant *Musa acuminata* Colla *acuminata* and double *balbisiana* (MA), *Musa* double *acuminata* cv. Pisang Mas (MAM), *Musa acuminata* cv. Pisang Awak (MAA), and *Musa* Basjoo Siebold (MBS), and *Musa* double *acuminata* and *balbisiana*-Prata (MAP) were investigated. Results exhibited that all starches exhibited high content of amylose (34.04–42.59%). According to the particle size, they were divided into medium (MA, MAM) (14.54–17.71 μm) and large (MAA, MBS, MAP) (23.01–23.82 μm) group. The medium group with A-type crystallization showed higher peak viscosity (PV), final viscosity, gel fracturability and gel hardness. For large group with B-type crystallization, the compact particle morphology, higher degree of crystallinity, short range order, gelatinization enthalpy, pasting temperature, lower porosity, water absorption capacity (WAC) and oil absorption capacity were found. In addition, the medium group with higher PV and gel hardness could be used as food thickening or gelling agents. The large group with higher Rc, short-range order, lower porosity and WAC could be potential to become raw material for resistant starch. All results showed the amylose content, had significant effect on the microstructure and physicochemical properties of starch samples. Outcomes in this investigation might provide a basis of theoretical application for industrial food production.

## Introduction

Banana (*Musa* spp.) is a subtropical and tropical giant perennial herb belonging to the Musaceae botanical family originating from Southeast Asia, Malaysia, and China. China is the second largest *Musa* producer after India (1, 2). Due to their low investment, high efficiency, and rapid income, bananas have become the fifth most important crop produced in the world after coffee, cereals, sugar, and cocoa. Banana production is also a source of employment in many developing countries (2). The unripe banana flesh has <1% soluble sugar content, 2 g/100 g fresh weight of fiber, 5.55 mg/100 g of potassium, and 20–23% starch content (wet basis) (3).

*Musa* is also a climacteric fruit with respiration and fruit ripening is dependent on ethylene production; therefore, it cannot be stored for longer periods (4). Until now, a large number of unsalable bananas decay frequently, resulting in a wastage of resources. Since *Musa* fruit is rich in starch, converting some green (unripe) banana pulps into starch would provide a more stable storage form (1–2 years) and increase its generality and practicality (5). Due to its cyclic regeneration capability and low cost, starch is considered one of the three major nutrient substances in human beings and is widely applied in food products as a green alternative material (6). In addition, the Chinese mutant banana species, a novel potential resource of high amylose content starch, was cultivated in the South Subtropical Crops Research Institute, Chinese Academy of Tropical Agricultural Sciences. However, the physicochemical properties and crystalline characteristics of these new types of Chinese banana starch with a high amylose content have not been reported.

Starch granules are semicrystalline polysaccharides consisting of amylose with (1–4)-linked α-glucan and amylopectin with α-(1–4)-linked α-glucan with α-(1–6) branch points (7). Due to various packing patterns of amylose and amylopectin structures, diverse crystallinity properties and physicochemical properties have been identified (8). Chen et al. (9) found that a high amylose content within the crystalline region confers a high rate of amylose reassociation after gelatinization for jackfruit seed starch, leading to its high retrogradation rate of the crystal nucleus. This phenomenon could lead to a significant change in the quality and nutrition structure of starch-based food products during food processing. Therefore, starch with a high amylose content has become the hot spot of research so that it can be used as potential raw materials for food additives and human staple food. Zou et al. (10) reported that the high amylose content of yam starch resulted in high proportions of a double helix, and compact and ordered crystalline structures, leading to high thermal stability and low digestibility, which demonstrated that a high amylose content of yam starch could be considered the resource of a high-resistant starch content (10). The new type of banana starch with high amylose content in this study needed to be further studied to be utilized as a potential resource of food additives and resistant starch.

Therefore, this study aimed to characterize the crystal structure and physicochemical properties of high-amylose starch from five mutant banana species grown in China: *Musa acuminata* Colla *acuminata* and double *balbisiana* (MA), *Musa* double *acuminata* cv. Pisang Mas (MAM), *Musa acuminata* cv. Pisang Awak (MAA), *Musa Basjoo* Siebold (MBS), and *Musa* double *acuminata* and *balbisiana*-Prata (MAP). The proximate composition, physicochemical properties, and crystal characteristics were measured. Principal component analysis (PCA) was used to determine the correlation between each characteristic parameter. The results obtained from this analysis will contribute to food and non-food applications in the future.

## Materials and methods

### Materials

Unripe fruits of all banana species were collected from the agricultural land of the South Subtropical Crops Research Institute, Chinese Academy of Tropical Agricultural Sciences (Guangzhou, China). The banana species were obtained in three batches-−29 August 2021, 25 September 2021, and 30 October 2021—and each batch included 20 kg of each banana species.

### Starch preparation

The unripe mutant banana pulp was dried in a vacuum freeze dryer (50°C for 48 h) and ground into banana flour. The *Musa* flour (100 g) was mixed with 1 L of distilled water, and the mixed liquor was sieved through a 100-mesh filter cloth. The filtrate was centrifuged (4,000 × g, 15°C, 10 min), the precipitate was dissolved in 1 L of NaOH (0.2% w/v), and the solution was stirred (10 min) to remove soluble fibers. The mixed liquor was centrifuged at 4,000 × g for 10 min, and the supernatant was discarded. Then, the brown skin (exogenous impurities) was removed, and the starch sediment was repeatedly washed with distilled water until neutrality was achieved. The remaining samples were dried at 50°C for 24 h until the moisture content was <13 g/100 g and was then filtered through a 100-mesh sieve (7).

### X-ray diffraction analysis

X-ray diffraction (XRD) patterns were analyzed using an X-ray diffractometer (Bede XRD Di System, Durham, United Kingdom). A copper tube was used to measure starch samples (40 kV and 200 mA, Cu Kα radiation at 0.154 nm). The diffraction pattern was measured by a step length of 0.02°, a scattering slit width of 1°, a slit width of 0.02 mm, and scanning from 4 to 40° (2°) at a speed of 4°/min. Each sample was evaluated in triplicate. The relative crystallinity of different starch samples was calculated as described by Barros et al. (5).

### Proximate composition of isolated starch

The AOAC Official Methods of Analysis. 18th edn. Association of Official Analytical Chemists; Arlington, VA, USA: 2012 method was used to measure the contents of moisture, total starch, ash, lipid, and protein (11).

### Amylose content

The amylose content was quantified as mentioned by Li et al. (12). A measure of 1 ml of ethanol and 9 ml of 1 M NaOH were mixed with a 100 mg of starch sample (dry basis) and then boiled in a water bath. After cooling to 25°C, the mixed solution was diluted to 100 ml. A volume of 2.5 ml of this aliquot was added to 50 ml of 1 M I_2_-KI. The absorbance of the obtained mixture was then measured at 620 nm in an ultraviolet spectrophotometer (SPECORD 250 Plus, Analytik Jena AG, Jena, Germany).

### Pasting properties

For studying the pasting properties, 3 g of the starch sample was added to 25 ml of deionized water in an RVA container. The solution was stirred at 960 rpm/min for 10 s, followed by 160 rpm/min, and then was analyzed using a Rapid Visco Analyzer (RVA super 4, Newport Scientific, Australia). The corresponding viscosity characteristics was calculated by a Stander 1 program in this instrument (12). The samples were incubated at 50°C for 1 min and then heated to 95C at 6 °C/min. The starch paste was cooled to 50C at 6°C/min, maintained at 95°C for 5 min, and then kept at 50°C for 2 min. All measurements were performed in triplicate.

### Morphology and particle size distribution analysis of starch granules

The granule morphology of the starch samples was examined using a scanning electron microscope (SEM) (Phenom ProX, Phenom Company, The Netherlands). The starch samples were applied to a silver plate coated with a thin film of gold (10 nm) and then kept at an accelerating voltage of 15 kV. Then, the samples were examined using a polarized light microscope linked to a CCD camera, using which we observed Maltese crosses (Olympus BX51, Tokyo, Japan).

The size distributions corresponding to granules of the starch were measured using a Malvern Mastersizer 3,000 laser diffraction size analyzer following the method of Barros et al. (5) and Ren et al. (13).

### Water and oil absorption capacity

A measure of 1 gram of the starch sample was taken in a centrifuge tube and then 10 ml of distilled water or first-grade peanut oil was added to it, and the mixture was pre-weighed. The solution was mixed for 1 h in a vortex oscillator. The mixture was centrifuged (2,000 × g, 30 min, 25 °C), the supernatant was discarded, and the tube containing the pellet was weighed. The oil and water absorption capacities were expressed as percentages of water or oil absorbed by the starch samples (11).

### Bulk, true densities, and porosity

Bulk densities were measured by using the method of Zhang et al. (14), except that 5 g of the starch sample was used. The bulk densities were calculated as the ratio of the weight of the starch sample to the occupied volume. True density (T_d_) was measured using the liquid displacement method and calculated as follows:


(1) $$\begin{array}{l} T_{d}=\frac{WS}{X+W-Y} \end{array}$$

where W is the weight of the sample, S is the specific gravity of xylene, X is the weight of the bottle and xylene, and Y is the weight of the bottle, xylene, and the sample.

The porosity (P_f_) of the starch sample was calculated using the following formula:


(2) $$\begin{array}{l} p_{f}=\left(1-\frac{B_{d}}{T_{d}}\right)\times 100\% \end{array}$$

where B_d_ is the bulk density and T_d_ is the true density.

### Thermal properties

A differential scanning calorimeter (DSC-Q2000 TA Instruments, USA) equipped with a thermal analysis data station (TA Instruments, New Castle, DE, USA) was used to study the gelatinization parameters of thermal properties following the method of Zhang et al. (7). A total of 5 mg of dry sample was mixed with 10 mg of distilled water in a crucible, sealed, incubated at room temperature for 24 h, and then the mixture was heated from 10 to 100°C (10°C /min). Universal Analysis Program (TA Instruments) was used to calculate T_o_, the temperature at which the tangential line from the lower temperature side of the peak intersects with the baseline; T_p_, the temperature at the tip of the peak; T_c_, the temperature at which the tangential line from the high-temperature side of the peak intersects with the baseline; and ΔH_g_, the area under the peak bound by the baseline on the graph. Each sample was evaluated in triplicate.

### Fourier transform infrared (FTIR) spectrum

The short-range order was analyzed using a Nicolet 6700 Fourier transform near-infrared spectrometer (Thermo Fisher Scientific, USA) linked to a smart ITR attachment. The scanning times and resolution were 64 and 4 cm^−1^, respectively. The ratio of 1,047/1,022 cm^−1^ was recorded (15).

### Gel texture properties of starch

A texture analyzer (TA.XT*Plus*, Texture Technologies Corp., United Kingdom) equipped with a weight sensor (max 50 kg) was used to conduct a texture profile analysis (TPA) of starch gels prepared by the RVA. The Texture Expert Exceed version 1.0 program (Stable Micro Systems software) was used to record and analyze the texture properties of the gel. A P/36R cylinder probe was used to carry out the TPA pattern, and a P/0.5R cylinder probe was used to determine the gel 0.5 pattern according to the methods in the program with 40% strain, a pre-test speed of 1.0 mm/s, a test speed of 2 mm/s, and a post-test speed of 2 mm/s. The hardness, fracturability, adhesiveness, springiness, cohesiveness, gumminess, chewiness, resilience, gel strength, gel rupture strength, gel rupture distance, and gel hardness of the sample were determined (16).

### Statistical analysis

Mean, standard deviations, and significant differences between values and correlations between parameters were calculated using SPSS 12.0.1 (SPSS Inc., Chicago, Illinois, USA). Significant differences between the means were determined by using Duncan's multiple range test at a significance level of 0.05. The significant differences, mean values, and standard deviation between values were identified by Duncan's multiple range tests at a significance level of 0.05 (SPSS 20.0., Inc., Chicago, Illinois, US). IBM SPSS 20.0 was used to analyze the principal component analysis (PCA), neural network analysis, and cluster analysis.

## Results and discussion

### Crystalline structure

**Figure 1:**
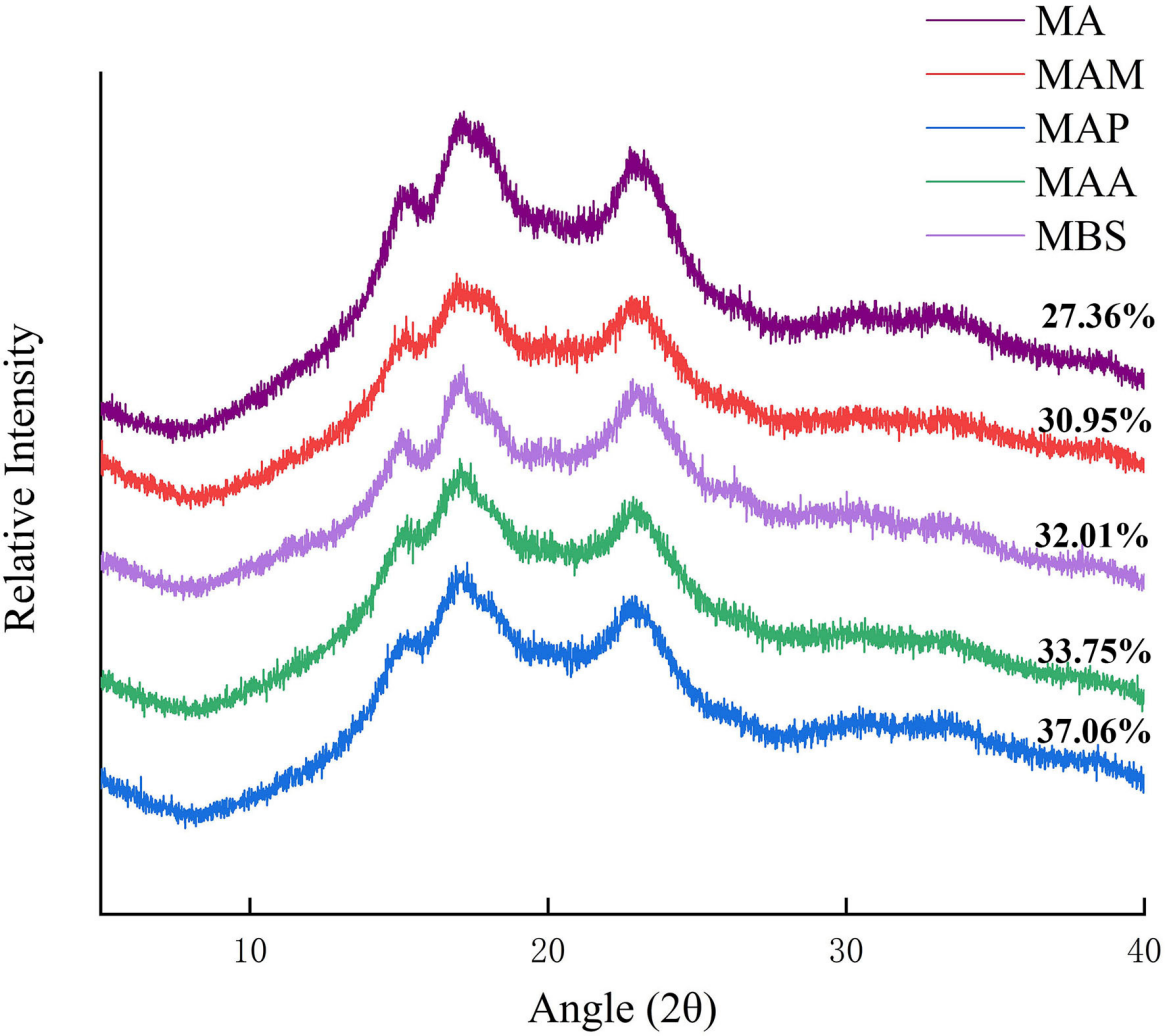
X-ray diffraction patterns of starch samples.

The characteristics of crystalline structures and long-range order in the starch granules were analyzed by XRD (Figure 1). The diffraction peaks of MA and MAM occurred at 2θ = 15, 17, 18, and 23°, which represent an A-type crystal structure, as mentioned in Jiang et al. (2). The MAA, MBS, and MAP occurred at 2θ = 15°, 17°, and 23°, indicating a B-type crystal structure (Figure 1), as describes by Zhang et al. (8). This result was broadly consistent with Utrilla-Coello et al. (17), who also found a B-type crystal structure for Mexico banana starch. The degree of relative crystallinity (R_c_) followed the order: MAP (37.06%) > MBS (33.75%) > MAA (32.01%) > MAM (30.95%) > MA (27.36%) (Table 1). A significant difference in the Rc value between banana samples was observed (p < 0.05). Based on Zou et al. (10), this diversity might be ascribed to the difference in the Bragg diffraction distance and characteristic size of the crystallite unit. Compared with the B- or C-type crystal structure of banana starch reported in a previous study (5), it was found that the type of the crystal structure and Rc of banana starch in the present study significantly differed. This distinct conclusion might be explained by the difference in the planting genotype, which causes a diversity of banana starch-branching enzyme (BE)IIb, gene-encoding starch synthase (SS)IIa, and granule-bound starch synthase (GBSS)I (15). The R_c_ of the A-type crystal structure banana starch samples was also higher than that of Enano, Morado, Valery, and Macho banana species starch samples (28–30%) (17). According to Bi et al. (18), a more ordered arrangement of amylopectin double helices in the semicrystalline lamellae is correlated with higher R_c_ values. Meanwhile, Zhang et al. (16) also reported that higher crystallinity (R_c_) is consistent with longer single helices of amylose and greater disruption of double helix crystals.

**Table 1:** Chemical composition, granule size and distribution, bulk, true density, porosity, and water and oil absorption capacity of starch samples isolated from five different banana species.

**Starch samples**	**MA**	**MAM**	**MAA**	**MBS**	**MAP**
Starch yields (%)	19.82 ± 0.35^cd^	23.61 ± 0.68^a^	21.14 ± 1.44^bc^	18.70 ± 0.98^d^	22.45 ± 1.22^ab^
Moisture (%)	6.67 ± 0.75^b^	3.88 ± 0.34^d^	5.94 ± 0.63^c^	10.28 ± 0.35^a^	3.51 ± 0.24^d^
Starch (%, db)	98.94 ± 0.24^a^	98.56 ± 0.76^a^	99.14 ± 0.65^a^	96.75 ± 0.59^b^	99.07 ± 0.36^a^
Protein (%, db)	0.39 ± 0.03^c^	0.54 ± 0.02^a^	0.22 ± 0.03^d^	0.47 ± 0.05^b^	0.44 ± 0.04^bc^
Lipid (%, db)	0.40 ± 0.05^c^	0.57 ± 0.03^b^	0.26 ± 0.02^c^	2.31 ± 0.16^a^	0.29 ± 0.03^c^
Ash (%, db)	0.27 ± 0.07^cd^	0.32 ± 0.05^bc^	0.39 ± 0.04^ab^	0.47 ± 0.04^a^	0.20 ± 0.03^d^
Amylose content (%, db)	40.58 ± 0.33^b^	41.09 ± 0.38^b^	41.39 ± 0.17^b^	34.04 ± 0.69^c^	42.59 ± 0.96^a^
Rc (%)	27.36 ± 0.18^e^	30.95 ± 0.47^d^	32.01 ± 0.40^c^	33.75 ± 0.56^b^	37.06 ± 0.71^a^
D [4,3]-D [3,2] (μm)	4.44 ± 0.55^b^	3.57 ± 0.52^b^	3.43 ± 0.76^b^	4.39 ± 0.69^b^	8.64 ± 0.71^a^
Bulk density (g/ml)	0.73 ± 0.04^d^	0.80 ± 0.03^cd^	0.88 ± 0.03^bc^	0.99 ± 0.03^ab^	1.02 ± 0.04^a^
True density (g/ml)	1.33 ± 0.08^b^	1.43 ± 0.06^b^	1.54 ± 0.08^ab^	1.70 ± 0.07^a^	1.71 ± 0.04^a^
Porosity (%)	45.28 ± 0.61^a^	44.13 ± 0.45^b^	42.67 ± 0.55^c^	41.50 ± 0.52^d^	40.24 ± 0.41^e^
Water absorption capacity (%)	88.45 ± 1.54^a^	83.20 ± 0.63^b^	80.92 ± 1.06^c^	77.84 ± 1.21^d^	74.53 ± 0.92^e^
Oil absorption capacity (%)	67.66 ± 0.35^a^	65.49 ± 1.03^b^	62.19 ± 1.31^c^	60.39 ± 0.32^d^	58.85 ± 0.76^e^

The Dx (10), Dx (50), and Dx (90) are the granule sizes at which 10, 50, and 90% of all the granules by volume are smaller. The D [3,2] and D [4,3] are the area mean diameter and volume mean diameter, respectively. Samples with different letters in the same column are significantly different at P < 0.05.

### Composition analysis

The moisture and protein contents of starch from all banana species were below 13 and 1.0%, respectively, in accordance with the food industry of China and Food Chemicals Codex (19), which required that the maximum value of moisture and protein were lower than 18% and 1% for the high purity starch (14). The amylose content of MA, MAM, MAA, MBS, and MAP was in the range of 34.04–42.59% (Table 1); therefore, all species tested were regarded as starch with a high amylose content according to published research (8). The amylose content of banana starch was partly higher than that reported by Bi et al. (18), who reported that the amylose content of MAA Cavendish starch was 31.41%. The difference in the amylose content of MAA may be due to various growing conditions. It was suggested that starch with a high amylose content may be used as a water-insoluble dietary fiber for promoting colony-balanced microbiota in the human small intestine (10).

According to Jiang et al. (2), the total starch content in some Chinese banana cultivates is about 92.10%, which is lower than that observed in this study (96.75–99.07%) (Table 1). Significant variations between the species were observed in starch yield, moisture, purity, protein, lipid, and ash amounts. In our study, moisture, protein, and lipid contents of the starch samples were compared with those of Colombian banana species (7.5–7.8%, 0.8–1.1%, and 0.1–0.8%) (20), but the protein content was lower in our study samples. MBS showed the highest ash and lipid content, a relatively high protein content, and the lowest purity and amylose content. Based on Zou et al. (10), the lower starch purity of MBS might be explained by the lower amylose content of MBS than the others, which caused a higher surface tension of MBS resulting in its stronger suction force of nano-surface. This showed that MBS was easily mixed with small bran-containing protein, ash, and lipids and was difficult to completely remove.

### Morphology and size distribution

**Figure 2:**
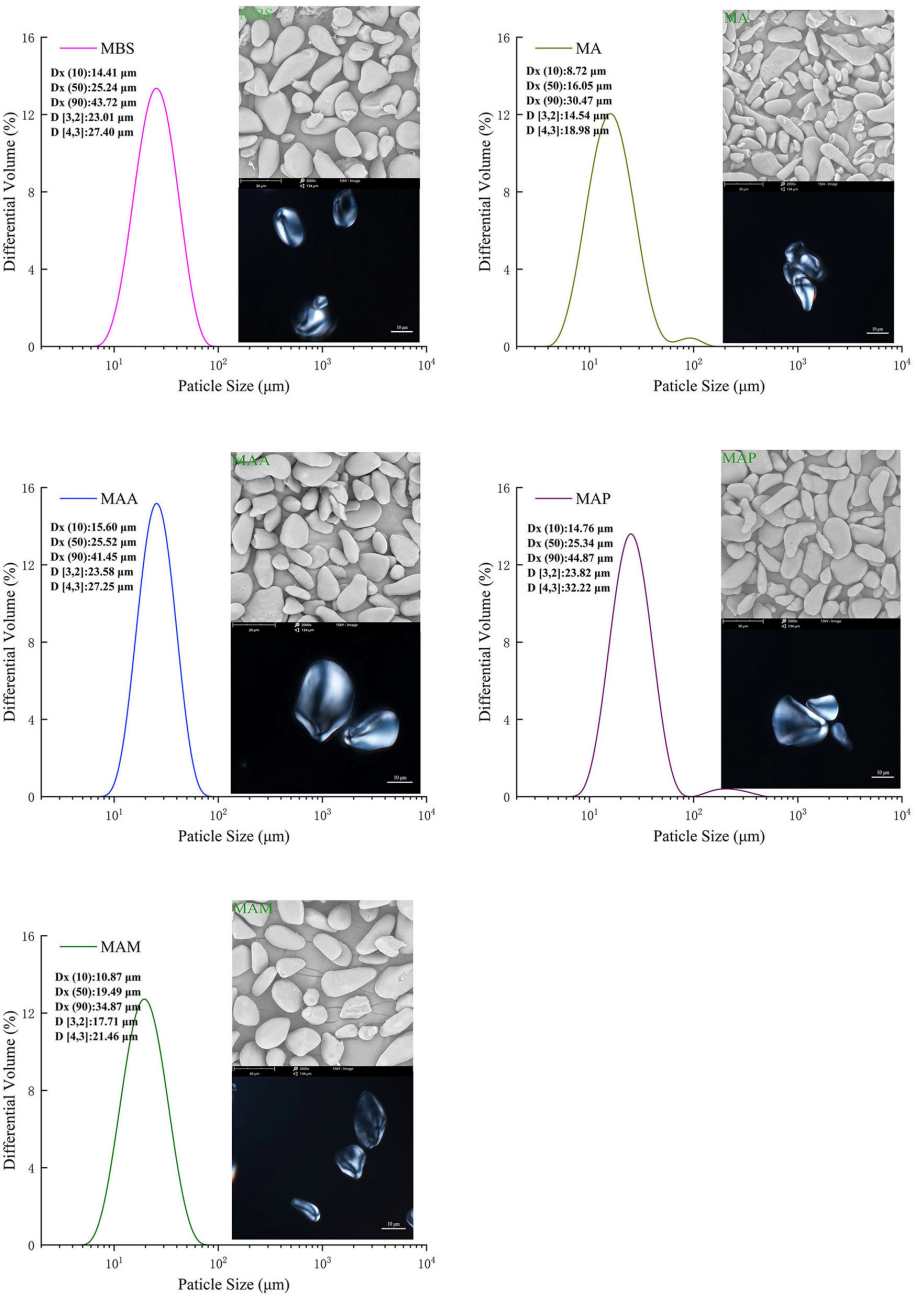
Particle size and distribution, and microscopy of starch granules at 2,000 × magnification (SEM) and visualized with a polarized light microscope.

The particle morphologies of MA, MAM, MAA, MBS, and MAP starch samples showed two major shapes: elongated and spherical (Figure 2 left). MA had consistent rod-like shapes with narrower and longer elongations, with a rough appearance compared with the other samples. Meanwhile, the starch granules of the MAM, MAA, MBS, and MAP species were flattened, and the surfaces were smooth. Our results were different from those of jackfruit seed starch samples, which had oval/bell shapes with a smoother granule surface (11). The differences may be ascribed to differences in amylose content and the type of the X-ray crystal. In addition, previous research (21, 22) revealed that a large number of smaller spherical clusters polymerized by amylopectin nanomodules within semicrystalline lamella could arrange beneath the surface of starch granules. This might have led to the lower root mean square roughness of the starch nanosurface and could also be used to explain the smoother granule surface of the MAM, MAA, MBS, and MAP than that of MA. Typical Maltese crosses (radial organization) were observed in the top position microstructures of all starch granules (Figure 2 right). This study showed that all samples have a semicrystalline structure, which is similar to the findings reported by Tongdang (23). In addition, the size distribution corresponding to the starch particle determined by the SEM is shown in Figure 2. For this part, Dx (10), Dx (50), and Dx (90) represent a starch particle size smaller than the corresponding diameter, which accounted for 10, 50, and 90% of the total amount of the particles, respectively (13). The Dx (10), Dx (50), and Dx (90) of MA, MAM, MAA, MBS, and MAP were in the following range: 8.72–30.47, 10.87–34.87, 15.60–41.45, 14.41–43.72, and 14.76–44.87 μm, respectively. The particle size distribution in this study was consistent with previous studies on banana (7.6–49.6 μm) (24) and yam starch (19.79–32.25 μm) (10). A surface area moment mean diameter (D[3,2]) and volume moment mean diameter (D[4,3]) were 18.98–32.22 μm and 14.54–23.82 μm, respectively, which were significantly different from the size distributions of the five banana starch samples (*p* < 0.05).

According to their significant area mean particle size (*p* < 0.05), the starch samples could be divided into two groups: medium (MA, MAM) and large (MAA, MBS, MAP) (Figure 2). MAP had significantly higher Dx (10), Dx (50), Dx (90), D[3,2], and D[4,3] values (14.76, 25.34, 23.82, 32.22, and 44.87 μm) (*p* < 0.05) than the other starch samples. Meanwhile, MA showed the lowest Dx (10), Dx (50), Dx (90), D [3,2], and D [4,3] values (8.72, 16.05, 30.47, 14.54, and 18.98 μm). A previous report showed that when the amylose content of banana starch increased from 26.54 to 29.01%, the granule sizes increased from 36.58 to 41.88 μm (5). It is suggested the difference in the particle size distribution among MA, MAM, MAA, MBS, and MAP may be due to the varieties of bananas and different plant growth conditions, leading to different amylose contents between each starch sample. Moreover, according to Ao and Jane (21) and Espinosa-Solis et al. (22), the highest particle size distribution for MAP might also be explained by that MAP showing the highest branching degree of amylopectin and the highest value of the degree of polymerization of trans-lamellar amylopectin long chains than the other samples. The difference values in D[4,3]-D[3,2] of MA, MAM, MAA, and MBS were comparable (Table 1), indicating that these starch samples had the highest granule consistency, while MAP had the lowest consistency, which agrees with Zou et al. (10). According to previous studies (5, 21), the surface micro-textures of starch granule including channel pores (extend into the hilum), emulsion bumps, fractal dimension, and roughness could significantly affect the contact area between the exposed hydroxyl group and free water. Therefore, morphology and size distributions are related to the properties of banana starch, and the physicochemical properties were measured as follows.

### Bulk, true density, porosity, and water, and oil absorption capacity

The large group (MAA, MBS, and MAP) had a significantly higher bulk density and true density than the medium-sized group (MA and MAM), and significant differences in bulk density were also found between samples in the medium-sized group (Table 1). The true densities of all species were similar to those of different rice starch samples (1.620–1.989 g/ml) (25); however, the bulk densities of all species were higher than those of different rice starch samples (0.633–0.675 g/ml) (26). MA showed the highest porosity (45.28%), followed by MAM (44.13%), MAA (42.67%), and MBS (41.50%), while the lowest porosity was found for MAP (40.24%). All these values were lower than those of different *Colocasia* starch samples (69.7–73.28%) (27). The differences in the density and porosity of the starch samples may be attributed to the particle size and granule morphology, which may be related to differences in starch composition, starch origin, and amylose contents (25, 27).

The water absorption capacity (WAC) and oil absorption capacity (OAC) of the five different samples were ranked as MA (88.45 and 67.66%) > MAM (83.20 and 65.49%) > MAA (80.92 and 62.19%) > MBS (77.84 and 60.39%) > MAP (74.53 and 58.85%) (Table 1), indicating that there was a significant difference between large- and medium-sized groups (*p* < 0.05). MA and MAP showed the highest and the lowest ability to bind water and oil, respectively. This differs from the WAC and OAC values of various rice starch samples (93.73–106.34% and 112.55–151.48%, respectively) (28). The different results between rice and banana starch may be due to distinct internal associative forces of water-binding sites within the starch molecule, according to Bhat and Riar (26). According to Falade & Christopher. (28), the lower water and oil binding ability of MAP particles might be because MAP particles have more ordinal and close-knit molecular structures.

### Thermal properties

**Figure 3:**
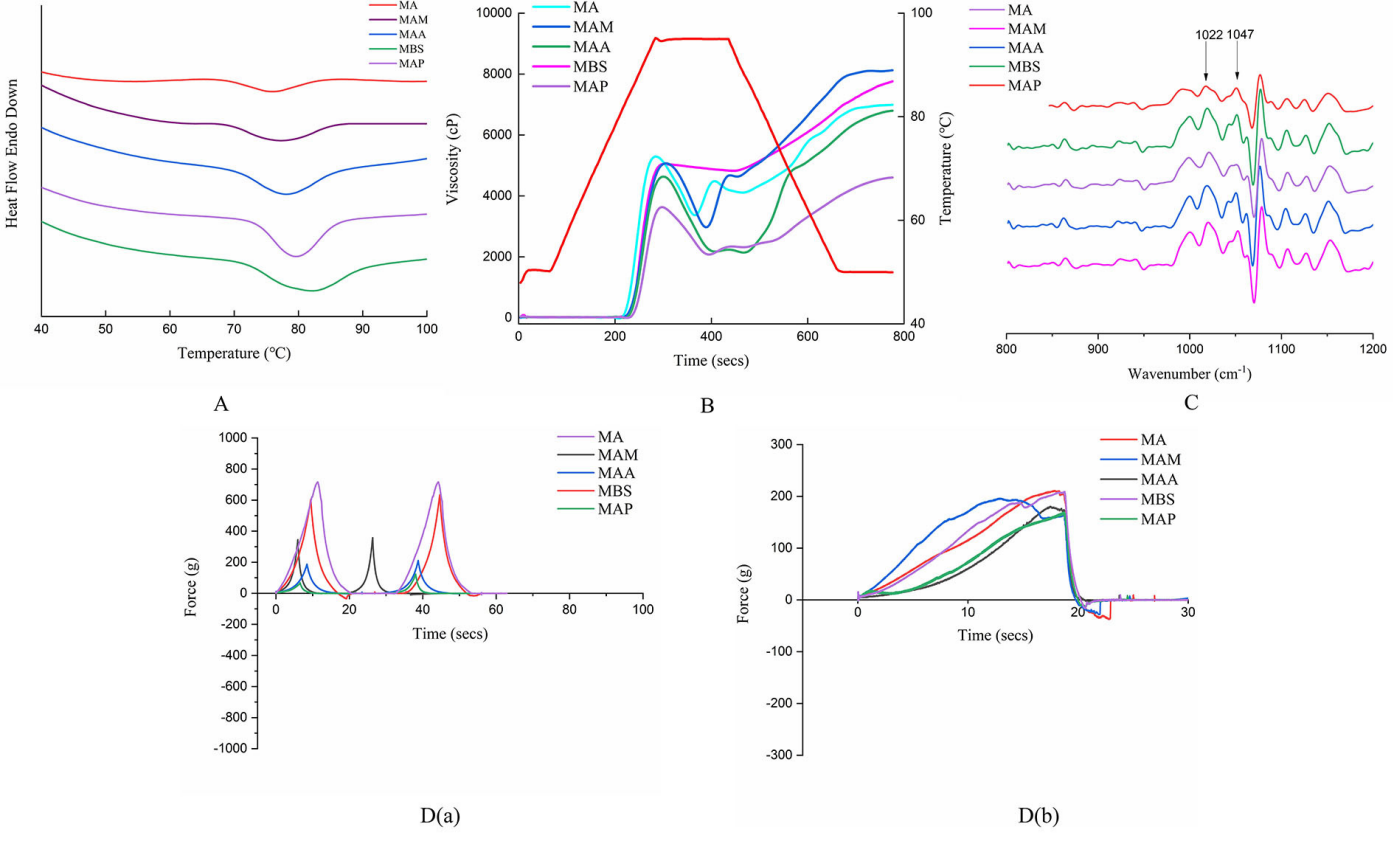
Physicochemical property of starch from five Chinese banana species. **(A)** Thermodynamic characteristics of starch samples. **(B)** Pasting profiles of different kinds of starch. **(C)** Deconvoluted FTIR spectra of starch samples. **(D)** Textural properties of starch gels from starch samples. a. Occlusal dynamic curve of TPA pattern. b. Occlusal dynamic curve of gel 0.5 pattern.

The significant thermal properties were determined for MA, MAM, MAA, MBS, and MAP (*p* < 0.05). Specifically, the onset temperatures (T_o_, 68.74–72.91°C) and conclusion temperatures (T_c_, 84.57–91.62°C) of MA, MAM, MAA, MBS, and MAP in this study (Figure 3A, Table 2) were similar to those of cassava starch (60.47and 79.32°C, respectively) (9), while the peak temperatures (T_p_, 76.08–82.55°C) and gelatinization enthalpies (ΔH_g_, 5.39 11.79 J/g) were consistent with those of Brazilian banana starch (70.58–72.17°C and 9.45–14.73 J/g, respectively) (5). T_p_ and ΔH depend on the amylose-to-amylopectin ratio and ordering of the double helix structure within crystalline regions (9). Moreover, T_o_, T_c_, and R remarkably correlated with the homogeneity and size of crystallites units, and the number of double helices and V-type polymorphs (27, 28). Therefore, the highest and lowest T_o_, T_p_, T_c_, R, and ΔH_g_ values in MAP and MA indicated that these samples showed the highest and lowest quantity of amylose contents and ordered helix structures of the starch crystallites, respectively, which is indicative of their degree of homogeneity and characteristic size.

**Table 2:** Thermal properties, relative crystallinity, short-range molecular order (1,047 cm^−1^/1,022 cm^−1^), pasting properties, and texture profiles of TPA pattern and gel 0.5 pattern of different starch samples.

**Starch samples**	**MA**	**MAM**	**MAA**	**MBS**	**MAP**
To (°C)	68.74 ± 0.53^d^	69.33 ± 0.60^cd^	70.61 ± 0.93^bc^	71.56 ± 0.57^b^	72.91 ± 0.89^a^
Tp (°C)	76.08 ± 0.96^d^	77.53 ± 0.82^c^	78.39 ± 0.74^bc^	79.22 ± 0.58^b^	82.55 ± 0.66^a^
Tc (°C)	84.57 ± 0.92^d^	87.78 ± 0.87^c^	86.91 ± 0.41^c^	89.86 ± 0.64^b^	91.62 ± 0.36^a^
ΔHg (J/g)	5.39 ± 0.37^a^	8.12 ± 0.43^c^	7.53 ± 0.21^c^	10.57 ± 0.35^b^	11.79 ± 0.49^a^
R (°C)	15.83 ± 0.72^c^	18.45 ± 0.73^b^	16.30 ± 0.67^c^	19.95 ± 0.77^a^	20.06 ± 0.53^a^
I 1047/1022	0.68 ± 0.02^a^	0.69 ± 0.03^a^	0.71 ± 0.04^ab^	0.76 ± 0.03^b^	0.87 ± 0.03^c^
Peak viscosity (cP)	5099 ± 1.78^b^	5399 ± 0.87^a^	4711 ± 1.55^d^	5051 ± 1.99^c^	3723 ± 2.54^e^
Trough viscosity (cP)	2747 ± 1.45^c^	3016 ± 1.35^b^	2027 ± 1.67^d^	4825 ± 2.44^a^	1962 ± 2.61^e^
Breakdown viscosity (cP)	2352 ± 1.27^c^	2383 ± 1.01^a^	2684 ± 1.95^b^	226 ± 2.69^e^	1761 ± 1.83^d^
Final viscosity (cP)	8161 ± 2.07^a^	6967 ± 1.99^c^	6815 ± 4.89^d^	7760 ± 9.82^b^	4623 ± 4.01^e^
Setback viscosity (cP)	5414 ± 1.39^a^	3951 ± 2.01^c^	4788 ± 2.04^b^	2935 ± 2.09^d^	2661 ± 2.22^e^
Peak time (mins)	5.07 ± 0.08^a^	4.73 ± 0.11^d^	5.00 ± 0.02^b^	5.07 ± 0.02^a^	4.93 ± 0.03^c^
Pasting temperature	82.25 ± 0.17^e^	83.55 ± 0.19^d^	84.00 ± 0.04^c^	85.15 ± 0.03^b^	86.25 ± 0.03^a^
Hardness	434.60 ± 3.39^a^	288.69 ± 6.54^c^	209.55 ± 1.98^d^	357.89 ± 5.17^b^	122.90 ± 2.33^e^
Fracturability	8.77 ± 0.29^b^	10.80 ± 0.22^a^	8.53 ± 0.03^c^	8.77 ± 0.10^b^	7.75 ± 0.05^d^
Adhesiveness (g.s)	−4.16 ± 0.04^d^	−13.40 ± 0.03^e^	−4.47 ± 0.07^c^	−0.63 ± 0.05^b^	−0.54 ± 0.03^a^
Springiness	0.95 ± 0.03^d^	1.07 ± 0.02^c^	1.19 ± 0.05^a^	1.11 ± 0.02^ab^	1.10 ± 0.02^ab^
Cohesiveness (g)	0.52 ± 0.05^e^	1.09 ± 0.01^b^	1.06 ± 0.03^bc^	1.44 ± 0.04^a^	1.02 ± 0.01^d^
Gumminess (g)	227.26 ± 1.97^c^	315.25 ± 6.61^b^	221.99 ± 1.89^d^	176.67 ± 2.63^e^	365.38 ± 1.59^a^
Chewiness (g)	216.57 ± 3.33^d^	336.17 ± 2.57^b^	263.34 ± 2.77^c^	196.11 ± 3.15^e^	402.11 ± 4.75^a^
Resilience (g)	0.28 ± 0.02^e^	0.71 ± 0.01^b^	0.76 ± 0.04^ab^	0.42 ± 0.01^d^	0.64 ± 0.02^c^
Gel strength (g)	58.40 ± 1.99^e^	96.83 ± 1.24^a^	78.20 ± 1.88^b^	19.49 ± 0.33^d^	19.96 ± 0.05^cd^
Gel rupture strength (Hardness) (g)	210.96 ± 5.60^c^	196.24 ± 2.39^b^	180.35 ± 7.32^d^	198.67 ± 3.10^ab^	169.16 ± 6.37^e^
Gel Rupture Distance (g)	14.29 ± 0.44^b^	10.29 ± 0.05^d^	13.99 ± 0.07^c^	9.60 ± 0.71^e^	15.00 ± 0.09^a^

To, onset temperature; Tp, peak temperature; Tc, conclusion temperature; R, gelatinization temperature range; ΔHg, enthalpy of gelatinization (J/g dry starch). Samples with different letters in the same column are significantly different at P < 0.05.

### Pasting properties

Significant variations were found in pasting behaviors among the five starch samples (Figure 3B, Table 2) (*p* < 0.05). The peak viscosity (PV, 3,723–5,399 cP) and breakdown viscosity (BDV, 226–2,383 cP) of banana starch in our study were comparable with those reported in previous studies on cassava, corn, potato, maize, wheat, and rice (PV, 1,852–8,046 cP and BDV, 767–6,717 cP) (12). Similarly, our trough viscosity (TV, 1,962–4,825) was similar to the TV values of different rice starch samples (1,635–3,403 cP) (26). MAM showed the highest PV compared with the other samples, indicating it has the most rigid gel network, which was shown to be formed during starch retrogradation. Meanwhile, MBS had the highest TV, which may have resulted from much lower swelling power and a comparatively smoother particle surface, according to Chen et al. (9). The highest BDV was found in MAA, indicating it has a much lower stability than the other samples under high temperatures and mechanical stirring based on Xia et al. (29).

The setback viscosity (SBV) and final viscosity (FV) of MA, MAM, MAA, MBS, and MAP were 2,661–5,414 cP and 4,623–8,161 cP, respectively (Figure 3B, Table 2), with the MA and MAP samples showing the highest and lowest FV and SBV values, respectively. Our range of values differs from a previous study on potato starch, showing an SBV of 202 cP and an FV of 1622 cP (29). These differences may be due to the length of a shorter amylopectin side chain and the amylose-to-amylopectin ratio (30). According to Chen et al. (9), granules with high FV (MA) have stronger reassociation forces (van der Waals) of amylose molecules during retrogradation. In addition, as described in a previous study (30), the higher SBV of MA than that of MAM, MAA, MBS, and MAP was responsible for its higher strength gel network formation. which involves amylose and amylopectin after gelatinization, which leads to a higher reassociation ability of MA amylopectin chains during cooling.

The peak time (4.93–5.07 min) and pasting temperature (Pt, 82.25to 86.25 °C) of the *Musa* starch samples varied significantly among samples (Figure 3B, Table 2). Our peak times were consistently lower than previous findings with rice starch (5.33–7.23 min), while the pasting temperatures (75.25–87.20°C) were comparable (Bhat & Riar, 2019). MAP showed the highest pasting temperature among the five starch samples, indicating its low porosity and high amylose content (Table 2) may result in a compact granular structure, attributed to stronger van der Waals forces between the amylose and amylopectin molecules based on the result of Zhang et al. (8).

### Short-range order analysis

The short-range order of the starch molecules was analyzed by infrared spectroscopy at 800–1,200 cm^−1^ (Figure 3C) with the absorbance ratios (1,047 cm^−1^/1,022 cm^−1^) of the five banana samples ranging from 0.68 to 0.87 (Table 2). A significant difference in short-range order value was observed for the five banana samples (*p* < 0.05). These results are comparable with those obtained for *Amaranthus* starch (0.644) (13). Starch molecules with high-amylose chain length distributions and alternately arranged order between the crystalline lamellae and the amorphous lamellae show a higher short-range order (15, 30). Moreover, the combination of the results of thermal properties and pasting properties suggested that the higher length of a shorter amylopectin side chain, higher amylose chain length distributions, and larger numbers of ordered amylopectin double helices within crystalline lamella might lead to higher short-range orders and compact granular structures (18), which contributed to the higher values of Pt, T_o_, T_p_, T_c_, R, and ΔH_g_. However, this resulted in lower FV, PV, and BDV due to the lower stability and strength of the gel network formed by weaker reassociation of amylose and amylopectin chains. These theories could be used to explain the differences in the short-range order, thermal properties, and pasting properties among the banana starch samples. Moreover, for a large size group with B-type crystallization, the overall higher amylose content, Rc, short-range order, and compact and smooth particle morphology could be attributed to higher ΔH_g_ and Pt. Based on published reports (27, 28), the denser molecular crosslinking network and the lower space distance of the six double helices in a crystal water unit cell led to a higher Rc, short-range order, and Pt, causing the higher RS content. Therefore, compared with a previous report on other banana starch samples, cassava, and rice starch (5, 28), a higher Rc, short-range order, and Pt were found for alarge-particle size group (MAA, MBS, MAP) (Tables 1, 2), which might be suggested the MA and MAM could be used as the potential raw materials of thickening or gelling agents to improve quality and taste during food processing.

### Textural properties

A texture profile analysis has been used to simulate human chewing *in vitro via* double occlusion. The apparent texture property parameter of gelatinous foods was measured by an A P/36R TPA pattern. In general, the internal texture property parameter is determined by using a P/0.5R cylinder probe. TPA using T/36 R and T/0.5 R probes showed consistent results, with the MA and MAP gels being the hardest (434.60 g for T/36R and 210.96 g for T/0.5R) and softest (122.90 g for T/36R and 169.16 g for T/0.5R), respectively, from the five samples [Table 2,Figure 3D (**a, b**)]. This differs from the hardness reported for wheat starch (68.0–131.1 g) (31). There is a positive correlation between gel hardness and the crystallization speed of the amylopectin double helix (31); therefore, the differences may be ascribed to variability in the amylose content and crystal structures of the two different starch sources. It was indicated that MA and MAP had the fastest and slowest amylopectin retrogradation, respectively.

MAP starch gels exhibited the greatest gel adhesiveness (−0.54 g.s), followed by MBS (−0.63 g.s), MA (−4.16 g.s), MAA (−4.47 g.s), and MAM (−13.40 g.s). This was lower than the values obtained for potato starch gel (−26.257 g.s to −81.315 g.s) (32), indicating that green banana starch might have a stronger starch gel network than potato. Previous results showed that gel adhesiveness is linked to R_c_ since the R_c_ of jackfruit seed starch increases from 13.29 to 15.87% when the gel adhesiveness increases from 134.38 to 121.31 g.s (16). Our results followed a similar trend, with MAP showing the highest R_c_ value, while MAM has the second lowest R_c_ value. Moreover, the lower gel adhesiveness of MAP is correlated with its higher speed of amylose retrogradation than that of the other samples, as reported by Nie et al. (32).

The ranges of fracturability, springiness, cohesiveness, gumminess, chewiness, resilience, cohesiveness, gel strength, gel rupture strength, and gel rupture distance were as follows: 7.75–10.80, 0.95–1.19, 0.52–1.44, 176.67–365.38, 196.11–402.11, 0.28–0.76, 19.49–96.83, 169.16–210.96, and 9.60–15.00 g, respectively [Table 2,Figure 3D (**a, b**)]. Higher fracturability, springiness, cohesiveness, gumminess, and chewiness but lower resilience of MA, MAM, MAA, MBS, and MAP were determined in this study compared with the earlier reports of wheat, potato, and rice starch (32, 33), indicating that green banana starch samples have stronger particle properties. The significant differences in the TPA between cereal starch and green banana starch samples may be due to the different amylose contents, and variations in true density, WAC, and short-range order. In addition, the medium-size group with A-type crystallization showed higher water absorption capacity and porosity in general, leading to their higher PV, FV, BDV, and gel hardness (Tables 1, 2). Compared with the obvious majority report for potato, wheat, and rice (29, 32, 33), the higher FV and gel hardness for the medium particle-size group (MA and MAM) indicated that MA and MAM could be used as the raw materials of thickening or gelling agents to improve quality and taste during food processing.

### Relationship between the physicochemical properties, amylose content, and particle size

#### Principal component analysis

**Figure 4:**
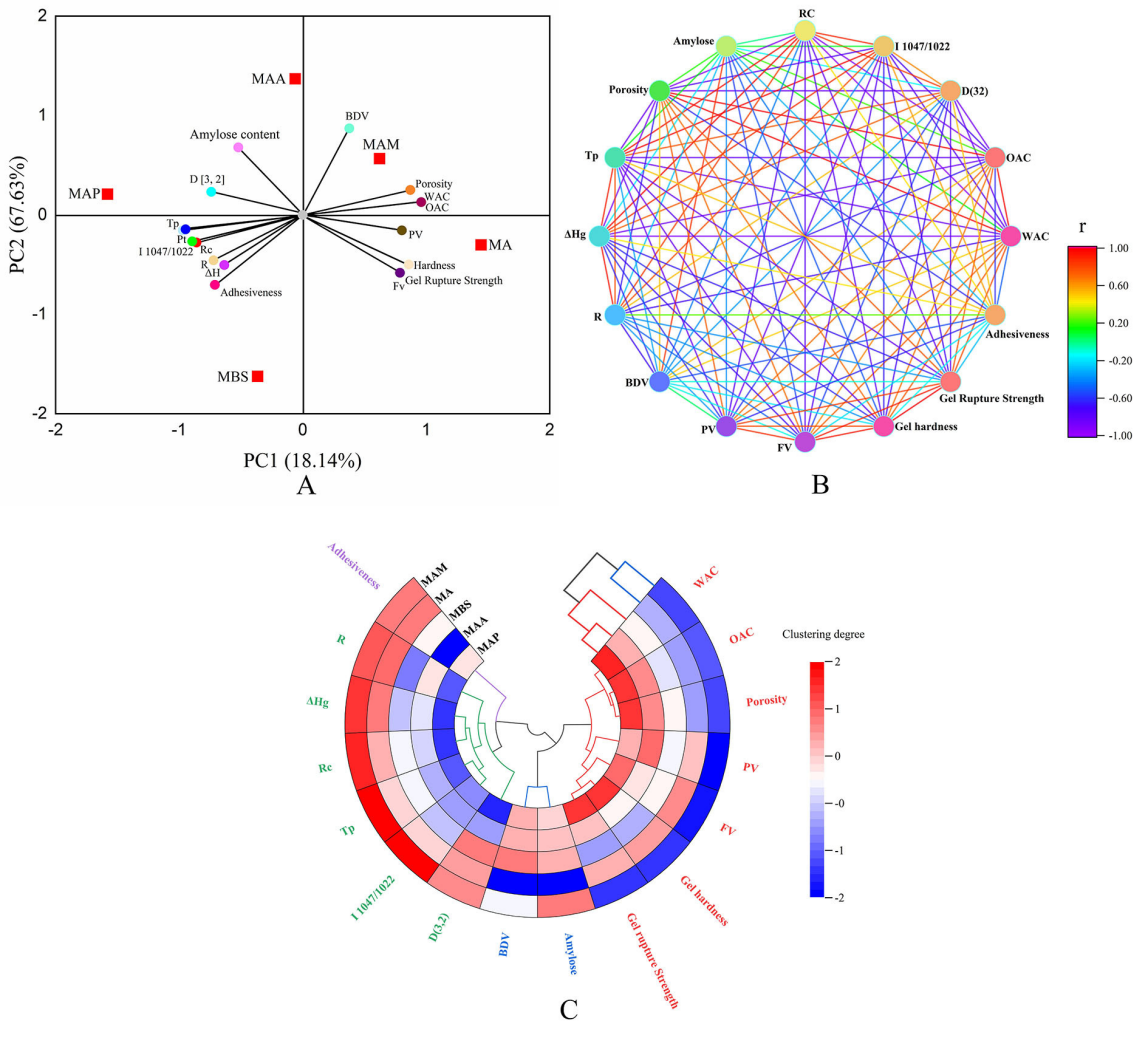
Statistical analysis. **(A)** Principal component analysis (PCA) score and loading plot of PC1 and PC2 of starch samples. **(B)** Neural network analysis based on the Pearson method. **(C)** Cluster tree analysis.

The interactions among the physicochemical characteristics using PCA showed that the starch samples were widely scattered, indicating that the type of starch markable affected the physicochemical and functional characteristics (Figure 4A). According to a previous study (15), these diversities might be explained by plant origins, climate, and environmental surroundings, leading to the distinct properties of starch self-assembled synthases, including synthase (BE)IIb, synthase (SS) IIa, and synthase I (GBSSI). As displayed in the PCA Figure, a significant positive correlation (*p* < 0.05) could be conjectured when the inclined angle between two components was remarkably smaller than 90°. Furthermore, there might be a positive correlation when the angle between the two components was remarkably higher than 90° (*p* < 0.05), whereas no correlation could be conjectured when the angle between the two components was nearly 90° (*p* > 0.05). The first principal component (PC1) mainly included PV, BDV, porosity, WAC and OAC, hardness, gel rupture strength, and FV. The second principal component (PC2) mainly included amylose content, R_c_, T_p_, Pt, ΔH_g_, R, D[3,2], and 1,047/1,022. A significantly positive correlation was observed between gel adhesiveness, R_c_, T_p_, Pt, ΔH_g_, R, D[3, 2], 1,047/1,022, and amylose content (*p* < 0.05), and among PV, porosity, WAC and OAC, hardness, gel rupture strength, and FV. The PV, BDV, porosity, WAC, and OAC also showed a positive correlation. These findings broadly correlate with the results reported in a study by Utrilla-Coello et al. (17), who found that when the T_p_ of different types of banana starch samples increased from 70.2 to 78.7°C, the ΔH increased from 10.4 to 15.1 J/g. Also, when the WAC of jackfruit seed starch increased from 88.98% to 112.46, the BDV increased from 174 to 981 cP (11). Overall, the proportions of gel adhesiveness, amylose, R_c_, T_p_, Pt, ΔH, R, D[3,2], and 1,047/1,022 were significantly negatively correlated with PV, porosity, WAC and OAC, hardness, gel rupture strength, and FV (*p* < 0.05). A similar result using cocoyam starch found that when the FV increased from 189.79 to 252.17 RVU, Pt decreased from 88.75 to 84.83°C (34). The amylose content did not correlate with adhesiveness and ΔH (*p* > 0.05). A proportion of BDV showed a weak correlation with the gel rupture strength, hardness, and FV (*p* > 0.05).

#### Neural network and cluster analysis

The neural network and cluster analysis based on the Pearson correlation are shown in Figures 4B,C, which could be used to further analyze the interactions among the physicochemical characteristics (8). As shown in Figure 4B, it was found that when the line showed deep red and deep purple between physicochemical characteristic parameters, it represents that there is a significant correlation between them (*p* < 0.5), while lines of other colors represented a less correlation between physicochemical characteristic parameters. In this part, a remarkably positive correlation was shown for Rc, Tp, Pt, ΔHg, R, D[3,2], and 1,047/1,022. Porosity showed a significantly positive correlation with WAC and OAC. FV was positively correlated with hardness, gel rupture strength, and PV and BDV. The amylose content showed a positive correlation with BDV. Rc displayed a negative correlation with WAC and OAC, FV, hardness, gel rupture strength, and PV. Porosity showed a significantly negative correlation with Rc, Tp, 1,047/1,022, and D[3,2]. Tp and 1,047/1,022 showed a negative correlation with WAC and OAC, FV, gel rupture strength, and PV. ΔHg showed a negative correlation with WAC and OAC, and D[3,2] displayed a significantly negative correlation with WAC and OAC, hardness, and gel rupture strength. The results of neural network analysis slightly differed from the results of principal component analysis. Based on Li et al. (12), this diversity might be due to the differences in statistical analysis mechanism. PCA relied on the kernel of optimal scaling and dimension reduction, and the neural network was based on the two-dimensional visualization of the correlation coefficient. Cluster analysis inferred that MAM and MA had relatively similar physicochemical characteristics. MBS, MAA, and MAP had cluster consistency. This conclusion was agreed with the classification for medium-size (MA, MAM) and large-size (MAA, MBS, MAP) size groups in this research. Moreover, four types of cluster trees were obtained. Rc, Tp, ΔHg, R, D[3,2], and 1,047/1,022 were found in one cluster tree. Porosity, WAC, OAC, FV, PV, hardness, and gel rupture strength were found in another cluster tree. PV and BDV appeared in one cluster tree. According to Zhang et al. (16), these phenomena indicated that these characteristic parameters could interact and affect each other. Adhesiveness showed weaker interactions with others. Zhang et al. (15) found similar results in jackfruit seed starch and reported that when Rc increased from 28.58 to 35.29%, Tp increased from 83.4to 86.2°C and 1,047/1,022 increased from 0.710 to 0.796.

The combination of PCA, neural network, and cluster analysis indicated that the crystalline structure, number of double helices, and double helix order, long-/short-range order (XRD/FTIR), amylose content, and particle size significantly influenced the physicochemical characteristics of A-type crystal green banana starch. Strong hydrogen bonds between the double helix of amylose and amylopectin form a higher long-/short-range order, double helix arrangement order, double helix structure content, and larger and smoother particle morphology (35), which resulted in stronger particle properties (higher R_c_, Pt, T_p_, and ΔH_g_, and lower WAC, FV, PV, gel hardness, and porosity) in MAP than the other banana starch samples, while the opposite particle properties were observed among the other samples. However, Zhang et al. (16) reported that the particle size negatively correlated with R_c_, Pt, T_p_, and ΔH_g_ in seed starch of different jackfruit species, indicating that small starch particles have stronger particle properties. Based on previous reports, the crystalline structure types and winding manner of amylose with single helices have important effects on the particle properties (17, 32, 33), which are likely to be relevant to the study of banana starch. In addition, compared with previous research studies (2, 5, 17, 18, 24), it was found that crystal structure type and amylose content of banana starch in the present study significantly differed, suggesting its higher starch extraction rate, Rc, semicrystalline conformation order, gelatinization enthalpy, gelation temperature, final viscosity, setback viscosity peak viscosity, breakdown viscosity, and gel hardness than banana starch of other species. Therefore, this phenomenon indicated that starch from Chinese mutant banana species might provide a wider range of applications for food or non-food products.

## Conclusion

The characteristics of starch from Chinese mutant banana species were investigated, which showed a high-amylose starch content. The crystal structure and physicochemical properties varied among the starch species (*p* < 0.05). Based on diverse starch properties, the starch samples were divided into the medium-size group (MA and MAM) and the large-size group (MAA, MBS, and MAP). The higher amylose content, Rc, short-range molecular order, Pt, Tp, and lower viscous characteristics and gel hardness were shown by the large-size group, and a contrary result was shown by the medium-size group. The A-type structure of banana starch was found in the medium-sized group, and the B-type structure was shown in the other group. Neural network and cluster analyses further showed that gel adhesiveness, R_c_, T_p_, Pt, ΔH, R, D[3,2], and 1,047/1,022 were significantly positively correlated (*p* < 0.05). A significant positive correlation was also exhibited among PV, porosity, WAC, and OAC; hardness, gel rupture strength, and FV; PV, BDV, porosity, WAC, and OAC. Meanwhile, gel adhesiveness, amylose, R_c_, T_p_, Pt, ΔH_g_, R, D[3,2], and 1,047/1,022 were significantly negatively correlated with PV, porosity, WAC and OAC, gel hardness, gel rupture strength, and FV (*p* < 0.05). These results demonstrated that a higher amylose content, short-range order, double helix arrangement order, double helix structure content, and larger and smoother particle morphology of MAP lead to its higher Pt, Tp, and ΔHg, and lower porosity, WAC, FV, PV, and gel hardness than those of MA, MAM, MAA, and MBS. These findings may be used as a reference to prompt a wider investigation of green banana starch for utilization in the food and non-food industries.

## Data availability statement

The original contributions presented in the study are included in the article/supplementary material, further inquiries can be directed to the corresponding author/s.

## Author contributions

CH: conceptualization, methodology, software, and validation. BL: formal analysis, investigation, writing—original draft preparation, and data curation. YZ: resources, project administration, funding acquisition, writing—review and editing, visualization, and supervision. All authors have read and agreed to the published version of the manuscript.

## Funding

This research was funded by the Key Research and Development Program of Hainan Province (ZDYF2022SHFZ122) and the Chinese Central Public-Interest Scientific Institution Basal Research Fund (1630142022007). This study was financially supported by the College of Light Industry and Food Engineering of Guangxi University and Spice and Beverage Research Institute, the Chinese Academy of Tropical Agricultural Sciences, and the Key Laboratory of Processing Suitability and Quality Control of the Special Tropical Crops of Hainan Province.

## Conflict of interest

The authors declare that the research was conducted in the absence of any commercial or financial relationships that could be construed as a potential conflict of interest.

## Publisher's note

All claims expressed in this article are solely those of the authors and do not necessarily represent those of their affiliated organizations, or those of the publisher, the editors and the reviewers. Any product that may be evaluated in this article, or claim that may be made by its manufacturer, is not guaranteed or endorsed by the publisher.

## Abbreviations

MA: *Musa acuminata* Colla *acuminata* and double *balbisiana*
MAM: *Musa* double *acuminata* cv. Pisang Mas
MAA: *Musa acuminata* cv. Pisang Awak
MBS: *Musa Basjoo* Siebold
MAP: *Musa* double *acuminata* and *balbisiana*-Prata
WAC: water absorption capacity
OAC: oil absorption capacity
Rc: relative crystallinity
PV: peak viscosity
BDV: breakdown viscosity
FV: final viscosity
SBV: back viscosity
TV: trough viscosity
PT: pasting temperature
To: onset temperature
Tp: peak temperature/gelatinization temperature
Tc: conclusion temperature
ΔHg: enthalpy of gelatinization
R: gelatinization range
TPA: textural profile analysis
PCA: principal component analysis.

## References

[1] Anyasi TA Jideani AIO Mchau GRA. Functional properties and postharvest utilization of commercial and non-commercial banana cultivars. Compr Rev Food Sci Food Saf. (2013) 12:509–22. 10.1111/1541-4337.12025 33412666

[2] Jiang H Zhang Y Hong Y Bi Y Gu Z Cheng L. Digestibility and changes to structural characteristics of green banana starch during *invitro* digestion. Food Hydrocoll. (2015) 49:192–9. 10.1016/j.foodhyd.2015.03.023

[3] Goswami B Borthakur A. Chemical and biochemical aspects of developing culinary banana (Musa ABB) “Kachkal”. Food Chem. (1996) 55:169–72. 10.1016/0308-8146(95)00072-0

[4] Aurore G Parfait B Fahrasmane L. Bananas, raw materials for making processed food products. Trends Food Sci Technol. (2009) 20:78–91. 10.1016/j.tifs.2008.10.003

[5] Barros Mesquita C Leonel M Franco CML Leonel S Garcia EL dos Santos TPR. Characterization of banana starches obtained from cultivars grown in Brazil. Int J Biol Macromol. (2016) 89:632–9. 10.1016/j.ijbiomac.2016.05.040 27180297

[6] Zhao Y Huang C Huang X Huang H Zhao H Wang S. Effectiveness of PECVD deposited nano-silicon oxide protective layer for polylactic acid film: Barrier and surface properties. Food Packag Shelf Life. (2020) 25:100513. 10.1016/j.fpsl.2020.100513

[7] Zhang Y Liu W Liu C Luo S Li T Liu Y. Retrogradation behavior of high-amylose rice starch prepared by improved extrusion cooking technology. Food Chem. (2014) 158:255–61. 10.1016/j.foodchem.2014.02.072 24731339

[8] Zhang Y Li B Xu F He S Zhang Y Sun L. Jackfruit starch: Composition, structure, functional properties, modifications and applications. Trends Food Sci Technol. (2021) 107:268–83. 10.1016/j.tifs.2020.10.041

[9] Chen J Liang Y Li X Chen L Xie F. Supramolecular structure of jackfruit seed starch and its relationship with digestibility and physicochemical properties. Carbohydr Polym. (2016) 150:269–77. 10.1016/j.carbpol.2016.05.030 27312638

[10] Zou J Xu M Wen L Yang B. Structure and physicochemical properties of native starch and resistant starch in Chinese yam (Dioscorea opposita Thunb). Carbohydr Polym. (2020) 237:116188. 10.1016/j.carbpol.2020.116188 32241403

[11] Zhang Y Zhu K He S Tan L Kong X. Characterizations of high purity starches isolated from five different jackfruit cultivars. Food Hydrocoll. (2016) 52:785–94. 10.1016/j.foodhyd.2015.07.037

[12] Li B Wang H Wang X Zhang Y Tan Y Zhang Y. Prediction of the postprandial blood sugar response estimated by enzymatic kinetics of in vitro digestive and fine molecular structure of artocarpus heterophyllus lam seed starch and several staple crop starches. Starch/Starke. (2019) 71:9–10. 10.1002/star.201800351

[13] Ren Y Guo K Zhang B Wei C. Comparison of physicochemical properties of very small granule starches from endosperms of dicotyledon plants. Int J Biol Macromol. (2020) 154:818–25. 10.1016/j.ijbiomac.2020.03.147 32198038

[14] Zhao J Zhang Y Wu Y Liu L Ouyang J. Physicochemical properties and in vitro digestibility of starch from naturally air-dried chestnut. Int J Biol Macromol. (2018) 117:1074–80. 10.1016/j.ijbiomac.2018.06.034 29890244

[15] Zhang Y Zuo H Xu F Zhu K Tan L Dong W. The digestion mechanism of jackfruit seed starch using improved extrusion cooking technology. Food Hydrocoll. (2021) 110:106154. 10.1016/j.foodhyd.2020.106154

[16] Zhang Y Hu M Zhu K Wu G Tan L. Functional properties and utilization of Artocarpus heterophyllus Lam seed starch from new species in China. Int J Biol Macromol. (2018) 107:1395–405. 10.1016/j.ijbiomac.2017.10.001 29017887

[17] Utrilla-Coello RG Rodríguez-Huezo ME Carrillo-Navas H Hernández-Jaimes C Vernon-Carter EJ Alvarez-Ramirez J. In vitro digestibility, physicochemical, thermal and rheological properties of banana starches. Carbohydr Polym. (2014) 101:154–62. 10.1016/j.carbpol.2013.09.019 24299760

[18] Bi Y Zhang Y Jiang H Hong Y Gu Z Cheng L. Molecular structure and digestibility of banana flour and starch. Food Hydrocoll. (2017) 72:219–27. 10.1016/j.foodhyd.2017.06.00334945445

[19] Committee on Food Chemicals Codex Food Chemicals Codex (FCC8). Natl Academy Pr, ISBN: 9780309088664.

[20] Chávez-Salazar A Bello-Pérez LA Agama-Acevedo E Castellanos-Galeano FJ Álvarez-Barreto CI Pacheco-Vargas G. Isolation and partial characterization of starch from banana cultivars grown in Colombia. Int J Biol Macromol. (2017) 98:240–6. 10.1016/j.ijbiomac.2017.01.024 28069347

[21] Ao Z Jane J Lin. Characterization and modeling of the A- and B-granule starches of wheat, triticale, and barley. Carbohydr Polym. (2007) 67:46–55. 10.1016/j.carbpol.2006.04.013

[22] Espinosa-Solis V Sanchez-Ambriz SL Hamaker BR Bello-Pérez LA. Fine structural characteristics related to digestion properties of acid-treated fruit starches. Starch/Staerke. (2011) 63:717–27. 10.1002/star.201100050

[23] Tongdang T. Some properties of starch extracted from three thai aromatic fruit seeds. Starch/Staerke. (2008) 60:199–207. 10.1002/star.200800641

[24] Peroni-Okita FHG Simão RA Cardoso MB Soares CA Lajolo FM Cordenunsi BR. In vivo degradation of banana starch: structural characterization of the degradation process. Carbohydr Polym. (2010) 81:291–9. 10.1016/j.carbpol.2010.02.022

[25] Das AB Goud V Das C. Microencapsulation of anthocyanin extract from purple rice bran using modified rice starch and its effect on rice dough rheology. Int J Biol Macromol. (2019) 124:573–81. 10.1016/j.ijbiomac.2018.11.247 30500502

[26] Bhat FM Riar CS. Effect of chemical composition, granule structure and crystalline form of pigmented rice starches on their functional characteristics. Food Chem. (2019) 297:124984. 10.1016/j.foodchem.2019.124984 31253275

[27] Deepika V Jayaram Kumar K Anima P. Isolation and partial characterization of delayed releasing starches of Colocasia species from Jharkhand, India. Carbohydr Polym. (2013) 96:253–8. 10.1016/j.carbpol.2013.04.002 23688478

[28] Falade KO Christopher AS. Physical, functional, pasting and thermal properties of flours and starches of six Nigerian rice cultivars. Food Hydrocoll. (2015) 44:478–90. 10.1016/j.foodhyd.2014.10.005

[29] Xia T Gou M Zhang G Li W Jiang H. Physical and structural properties of potato starch modified by dielectric treatment with different moisture content. Int J Biol Macromol. (2018) 118:1455–62. 10.1016/j.ijbiomac.2018.06.149 30170362

[30] Sandeep S Singh N Isono N Noda T. Relationship of granule size distribution and amylopectin structure with pasting, thermal, and retrogradation properties in wheat starch. J Agric Food Chem. (2010) 58:1180–8. 10.1021/jf902753f 20043631

[31] Sandhu KS Kaur M Mukesh. Studies on noodle quality of potato and rice starches and their blends in relation to their physicochemical, pasting and gel textural properties. LWT - Food Sci Technol. (2010) 43:1289–93. 10.1016/j.lwt.2010.03.003

[32] Nie H Li C Liu PH Lei CY Li J Bin. Retrogradation, gel texture properties, intrinsic viscosity and degradation mechanism of potato starch paste under ultrasonic irradiation. Food Hydrocoll. (2019) 95:590–600. 10.1016/j.foodhyd.2017.08.035

[33] Choi SG Kerr WL. Water mobility and textural properties of native and hydroxypropylated wheat starch gels. Carbohydr Polym. (2003) 51:1–8. 10.1016/S0144-8617(02)00083-8

[34] Falade KO Okafor CA. Physicochemical properties of five cocoyam (*Colocasia esculenta* and *Xanthosoma sagittifolium*) starches. Food Hydrocoll. (2013) 30:173–81. 10.1016/j.foodhyd.2012.05.006

[35] Ji X Yin M Hao L Shi M Liu H Liu Y. Effect of inulin on pasting, thermal, rheological properties and *in vitro* digestibility of pea starch gel. Int J Biol Macromol. (2021) 193:1669–75. 10.1016/j.ijbiomac.2021.11.004 34742552

